# Establishing a Reference Baseline for Midday Stem Water Potential in Olive and Its Use for Plant-Based Irrigation Management

**DOI:** 10.3389/fpls.2021.791711

**Published:** 2021-11-26

**Authors:** Ken Shackel, Alfonso Moriana, Giulia Marino, Mireia Corell, David Pérez-López, Maria Jose Martin-Palomo, Tiziano Caruso, Francesco Paolo Marra, Luis Martín Agüero Alcaras, Luke Milliron, Richard Rosecrance, Allan Fulton, Peter Searles

**Affiliations:** ^1^Department of Plant Sciences, University of California, Davis, Davis, CA, United States; ^2^Departamento de Agronomía, ETSIA, Universidad de Sevilla, Seville, Spain; ^3^Unidad Asociada al CSIC de Uso Sostenible del Suelo y el Agua en la Agricultura (Universidad de Sevilla-IRNAS), Seville, Spain; ^4^Departamento de Producción Agraria, CEIGRAM, Universidad Politécnica de Madrid, Madrid, Spain; ^5^Department of Agricultural, Food and Forest Sciences (SAAF), University of Palermo, Palermo, Italy; ^6^Agencia de Extensión Rural Aimogasta, Instituto Nacional de Tecnología Agropecuaria, Aimogasta, Argentina; ^7^University of California Cooperative Extension, Oroville, CA, United States; ^8^College of Agriculture, California State University, Chico, Chico, CA, United States; ^9^University of California Cooperative Extension, Red Bluff, CA, United States; ^10^Centro Regional de Investigaciones Científicas y Transferencia Tecnológica de La Rioja (CRILAR-Provincia de La Rioja-UNLaR-SEGEMAR-UNCa-CONICET), Anillaco, Argentina

**Keywords:** deficit irrigation, *Olea europaea*, stem water potential, vapor pressure deficit, baseline

## Abstract

Midday stem water potential (SWP) is rapidly becoming adopted as a standard tool for plant-based irrigation management in many woody perennial crops. A reference or “baseline” SWP has been used in some crops (almond, prune, grape, and walnut) to account for the climatic influence of air vapor pressure deficit (VPD) on SWP under non-limiting soil moisture conditions. The baseline can be determined empirically for field trees maintained under such non-limiting conditions, but such conditions are difficult to achieve for an entire season. We present the results of an alternative survey-based approach, using a large set of SWP and VPD data collected over multiple years, from irrigation experiments in olive orchards located in multiple countries [Spain, United States (California), Italy, and Argentina]. The relation of SWP to midday VPD across the entire data set was consistent with an upper limit SWP which declined with VPD, with the upper limit being similar to that found in *Prunus*. A best fit linear regression estimate for this upper limit (baseline) was found by selecting the maximum *R*^2^ and minimum probability for various upper fractions of the SWP/VPD relation. In addition to being surprisingly similar to the *Prunus* baseline, the olive baseline was also similar (within 0.1 MPa) to a recently published mechanistic olive soil-plant-atmosphere-continuum (SPAC) model for “super high density” orchard systems. Despite similarities in the baseline, the overall physiological range of SWP exhibited by olive extends to about −8 MPa, compared to about −4 MPa for economically producing almond. This may indicate that, despite species differences in physiological responses to low water availability (drought), there may be convergent adaptations/acclimations across species to high levels of water availability. Similar to its use in other crops, the olive baseline will enable more accurate and reproducible plant-based irrigation management for both full and deficit irrigation practices, and we present tentative SWP guidelines for this purpose.

## Introduction

Crop productivity is closely linked to crop water use (e.g., Howell, 1990) and improving the efficiency of water use in agriculture has been an ongoing focus of research worldwide (e.g., Velasco-Muñoz et al., 2018). For some woody perennial crops, reducing or eliminating irrigation during specific periods of development (e.g., Chalmers et al., 1981) has been shown to produce economically beneficial effects, such as an improved fruit drying ratio in prunes (Lampinen et al., 1995), decreased fruit drop in peach (Li et al., 1989), and increased control of hull rot disease in almonds (Teviotdale et al., 2001). Hence, these crops may be good candidates for deficit water management strategies to increase overall water use efficiency. In woody perennial crops, however, the effect of any given deficit irrigation regime can depend strongly on soil conditions (e.g., Lampinen et al., 1995). Hence, the plant-based approach of midday stem water potential (SWP; Shackel, 2011) has become a widely accepted tool for deficit irrigation management.

Olive (*Olea europaea* L.) is considered to be a drought resistant species (Connor, 2005) and also exhibits a wide range of SWP under cultivated conditions. However, olive also exhibits differential sensitivity of yield to SWP at different periods of crop development. Olive trees are an evergreen species most often grown in Mediterranean climate regions with shoot growth and bloom occurring during spring in mature orchards. Fruit set occurs as evaporative demand increases, with fruit growth and oil accumulation occurring under fairly high evaporative demand conditions in the summer and fall, and with harvest varying from the end of summer to early winter. In addition to occurring under different environmental conditions, all of these processes exhibit different levels of sensitivity to low SWP. Shoot growth and flowering are very sensitive to water limited conditions, and while these processes normally occur at a time in the season when soil water is not limiting, supplemental irrigation may be needed under drought conditions or in locations with delayed growth cycles (Moriana et al., 2003; Pérez-López et al., 2007). SWP of around −2 MPa reduced fruit size due to reduced endocarp growth (Gómez del Campo, 2013; Gómez del Campo et al., 2014) with more severe SWP deficits (−4 MPa at predawn) affecting bud development and reducing next season bloom (Gucci et al., 2019). Once endocarp growth finishes, the number of fruits is relatively constant and pit hardening occurs (Rapoport et al., 2013). After this phase, the sensitivity of yield to water stress is reduced (Goldhamer, 1999; Moriana et al., 2003; Fernández et al., 2013; Girón et al., 2015; Ahumada-Orellana et al., 2017; Corell et al., 2020). Even under very severe water stress conditions (SWP below −5 MPa) yield may only be slightly reduced, particularly if there is an adequate recovery in SWP before harvest (Moriana et al., 2003; Fernández et al., 2013; Ahumada-Orellana et al., 2017). Oil accumulation prior to harvest is usually coincident with autumn rains under Mediterranean climate conditions, but several authors have suggested that moderate water stress does not substantially reduce oil accumulation (Moriana et al., 2003; Lavee et al., 2007; Ben-Gal et al., 2021) and improves oil extractability. Reduction in oil accumulation is likely to occur only with SWP values consistently below −2 MPa (Hueso et al., 2019). Postharvest irrigation is not commonly studied, but Agüero-Alcaras et al. (2021) reported no significant differences in next season yield over a wide range of postharvest water stress conditions.

The above values provide some guidance for an allowable lower range of SWP, but from a practical as well as scientific standpoint it is important to understand this physiological range in the context of both upper and lower limits. McCutchan and Shackel (1992) were the first to propose SWP as a reliable physiological indicator of water stress, in part because a stable relation over much of the growing season was found between SWP and vapor pressure deficit (VPD) under non-limiting soil moisture conditions. This relation enabled a reliable prediction of SWP for a “fully irrigated state or condition” (i.e., from an irrigation perspective). In essentially all previous and many current irrigation studies, the highest irrigation level is simply assumed to be non-water-limiting. However, localized water application systems (e.g., micro-irrigation) create zones of wet and dry soil, and, particularly for woody perennials, there are typically roots present in both zones throughout the season. If roots in dry soil influence overall plant water relations, then irrigation at 100% of crop evapotranspiration (ET_*c*_) in the wetted soil zones may not establish a physiologically non-soil-water-limited condition for the plant. To the authors knowledge, other than the McCutchan and Shackel (1992) study in *Prunus*, there has only been one study in olive (Morales-Sillero et al., 2013) in which the entire soil volume was maintained at a high moisture content over the growing season. Morales-Sillero et al. (2013) measured leaf rather than SWP in olive trees, but also did not report any relation of water potential to VPD. A number of studies in olive have found a linear relation of SWP to VPD (Morales-Sillero et al., 2013; Corell et al., 2016, 2020; Martin-Palomo et al., 2020), indicating that a relationship exists, but may be influenced by a number of factors, potentially including unintended effects of dry soil areas.

Based on the principle that the majority of water movement in soils and plants is driven primarily by differences in water potential (e.g., Kramer and Boyer, 1995), it is expected that SWP will depend on a large number of independent physical and biological factors such as soil, root, and stem hydraulic properties as well as plant transpiration as determined by stomatal and atmospheric conditions. Hence, it is not clear that an upper limit to SWP should exist, that it should be largely independent of soil and tree conditions, and that it should have a reproducible relation simply to VPD. However, the experimentally determined upper limit reported by McCutchan and Shackel (1992) produced a robust estimate for an upper limit of SWP that was supported by further studies in both prune (Shackel, 2011) and almond (Shackel et al., 2010) orchards. The objective of the current study in olive was to determine if a large survey of SWP and VPD values from olive orchards in multiple countries might produce an upper limit reference SWP baseline.

## Materials and Methods

### Survey Sites and Measurements

Much of the data used for this survey study was obtained from previous publications, and Table 1 summarizes the locations and additional characteristics of each of the survey sites. All orchards were managed commercially and drip irrigated. The multi-year and multi-location studies provided a large data set with variable ranges in VPD and SWP. References are listed in Table 1 where further information on particular sites may be obtained. Five different table (Manzanillo, Noceralla de Belice, and Olivo di Mandanici) and oil (Arbequina and Cornicabra) cultivars were used in these experiments in Argentina, Italy, Spain, and United States (California). Most data were from Arbequina and Manzanillo cvs but in different locations and management systems. Orchard age ranged from 2 to more than 10 years-old, but most orchards would be considered mature based on yield. Only the youngest in Coria del Rio (Spain) and Sciacca (Italy) were orchards with less yield than mature conditions and could be considered young. Tree density varied from high density (HD; around 300–350 trees per ha) to super high density (SHD), hedgerow orchards (>1000 trees per ha).

**TABLE 1:** Description of the sites used in the survey.

Country	Site	References	GPS	Year	CV	AGE	Soil	Density	Use
Argentina	Aimogasta (La Rioja)	Correa-Tedesco et al., 2010	28°33′S, 66°49′W	2005–2007	Manzanillo	6	Loamy sand	8 × 4	Table
Argentina	Aimogasta (La Rioja)	Agüero-Alcaras et al., 2021	28°35′S, 66°42′W	2009–2010	Manzanillo	10	Loamy sand	8 × 4	Table
Argentina	Chilecito (La Rioja)	Unpublished	29°09′S, 67°26′W	2017–2018	Arbequina	4	Gravelly sand	4 × 1.5	Oil
Spain	Ciudad Real	Unpublished	39°N, 5°6′W	2012–2015	Cornicabra	14	Shallow clay loam	7 × 4.76	Oil
Spain	Coria del Rio (Seville)	Martin-Palomo et al., 2020	37°N, 6°3′W	2014–2016	Manzanillo	43	Sandy loam	7 × 5	Table
Spain	Dos Hermanas (Seville)	Corell et al., 2020	37°25′N,5°95′W	2015–2017	Manzanillo	30	Sandy loam	7 × 4	Table
Spain	Carmona (Seville)	Unpublished	37.5°N, 5.7°W	2017–2019	Arbequina	11	Sandy loam	4 × 1.5	Oil
Spain	Coria del Rio (Seville)	Unpublished	37°N, 6°3′W	2015	Manzanillo	2	Sandy loam	4 × 1.5	Table
Italy	Marsala	Marra et al., 2016	37°46′28″N, 12°30′19″E	2008–2009	Arbequina	4	Sandy clay loam	1.5 × 3.5	Oil
Italy	Sciacca	Marino et al., 2016, 2021	37°32′N, 13°02′E	2014–2015	Nocellara del Belice and Olivo di Mandanici	3–4	Sandy clay loam	5 × 3, 5 × 2, 7 × 7	Oil, table
United States (CA)	Genoa	Unpublished	39°54′16.04″N, 122°17′14.20″W	2011	Manzanillo	>10	Loam, gravelly loam	7.7 × 3.6	Table
United States (CA)	Haro	Unpublished	39°49′N, 122°23′W	2011	Manzanillo	>10	Gravelly loam, sandy loam	9.0 × 5.8	Table
United States (CA)	Nielsen	Unpublished	39°44′59.36″N, 122°8′51.97″W	2009, 2011	Manzanillo	6, 8	Sandy loam	3.6 × 5.5	Table

Stem water potential was typically measured over multiple years as part of irrigation experiments. SWP was determined on individual trees as described previously (Fulton et al., 2001). Briefly, a shaded leaf or short stem located near the main trunk within the tree canopy was covered with a reflective plastic bag for longer than 10 min (typically 1–2 h) to allow equilibration with the water potential of the stem at the point of attachment. A Scholander-type pressure chamber was then used to measure SWP. Olive trees are a Mediterranean species, typically growing under hot and dry summer and relatively warm winter conditions. However, even in these regions, climatic conditions can be very different. For instance, the experiments in Ciudad Real (central Spain, Pérez-López et al., 2007) are in a production zone with a shorter and cooler summer than that in Dos Hermanas (south Spain, Corell et al., 2020). In all experiments, hourly climatic data were measured with automated stations either at the experimental plots, or in nearby locations with the same environment as the experimental plots. Hourly, mid-afternoon climatic measurements were used to calculate hourly air VPD (Tetens, 1930) that coincided with the period of SWP measurement.

### Data Assumptions and Analysis

Based on the hypothesis that there may be a practical upper limit to SWP at a given level of VPD for trees under non-soil-water-limited conditions (i.e., the “baseline” relation of Shackel, 2011), a total of 837 (SWP, VPD) values over all sites, years, and experimental treatments, were divided into groups based on 0.5 kPa classes of VPD. Assuming that each class would contain SWP values that were at or near the upper limit (i.e., if rain or irrigation had resulted in non-soil-water-limited conditions for that site and date) as well as SWP values below this limit, a range of uppermost (least negative) fractions (0.02–0.16) of SWP and the corresponding VPD values were averaged, and the average points used in a regression analysis of SWP on VPD. Since there was only one average (SWP, VPD) point per VPD class, each fraction contained the same number of (SWP, VPD) points, so the uppermost fraction which exhibited the highest regression *R*^2^ and lowest probability was used as the best fit estimate of the non-water-limited (“baseline”) relation of SWP to VPD. All statistical analyses were conducted in SAS 9.4 (SAS institute, Cary, NC, United States).

### Comparison to Data From the Literature

First, the baseline estimate for olive was compared to that found in prune and almond (Shackel, 2011). Second, a set of SWP and VPD values obtained from a multi-compartment hydraulic model for olive under simulated non-soil-water-limiting conditions for two contrasting planting densities (García-Tejera et al., 2021) was kindly provided by the authors. The relation of SWP to VPD for this set of data was determined and compared to the baseline estimate for olive. Lastly, previously published data of leaf conductance (Gs) to SWP in almond (Spinelli et al., 2016) was also compared to data in olive as reported by Marino et al. (2018) and Ahumada-Orellana et al. (2019). The raw data for each was kindly provided by the authors and fitted using a smoothed spline function (Proc Transreg, SAS 9.4) in order to avoid any *a priori* assumptions regarding the functional form of the (Gs, SWP) relation.

## Results

### Relation of Stem Water Potential to Vapor Pressure Deficit

**FIGURE 1:**
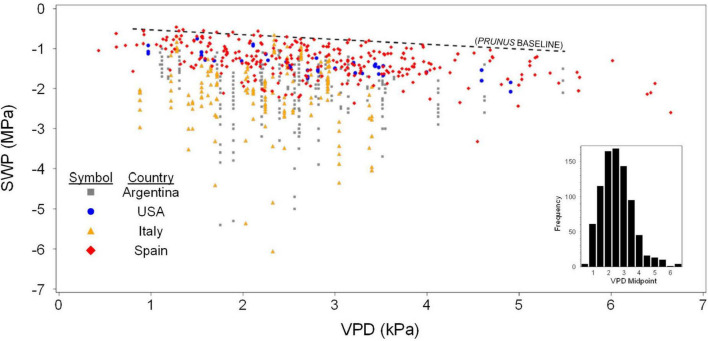
All survey data of SWP to midday VPD from four countries. Each point is an individual measurement, with vertical lines of points indicating SWP values that were collected at the same site and time. Also shown for reference is the baseline relation found for *Prunus* (dashed line). Equation for the *Prunus* baseline is SWP (MPa) = –0.12 × VPD – 0.41. Inset shows the number of SWP measurements associated with each 0.5 kPa class of VPD.

**FIGURE 2:**
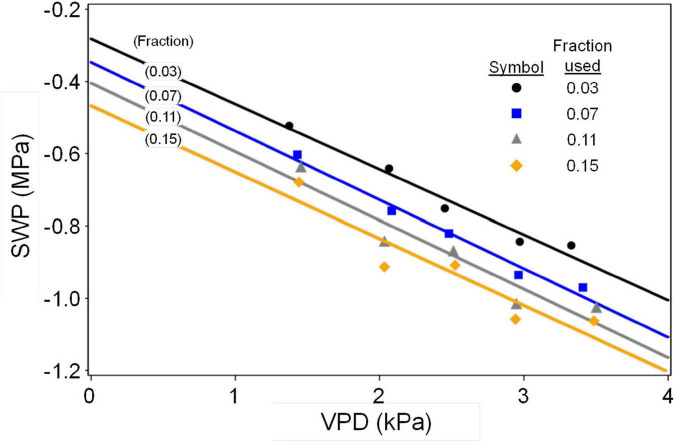
Relation of average SWP to average VPD for representative upper fractions of SWP values from 0.5 kPa classes of VPD. Also shown are the regression lines for each fraction. Only SWP data from the central 5 VPD classes (1.5–3.5 kPa midpoints) were used. Slopes were –0.17, –0.18, –0.19, and –0.18, and intercepts were –0.29, –0.34, –0.40, and –0.46, respectively for fractions of 0.03, 0.07, 0.11, and 0.15.

**FIGURE 3:**
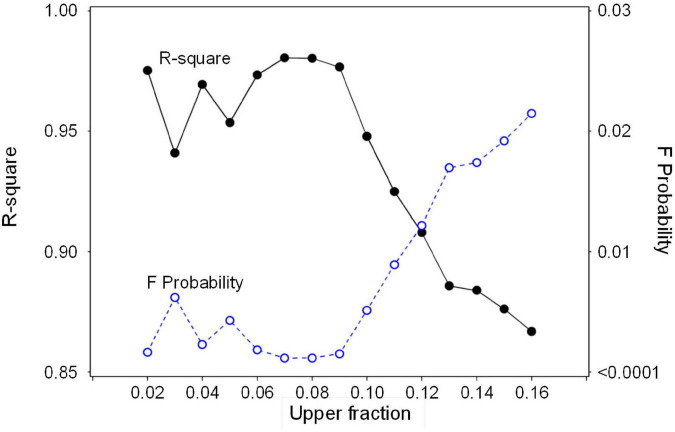
Regression statistics (R-square and F Probability) for the relation between average SWP and average VPD (as in Figure 2) for a range of upper fractions of SWP values.

Olive SWP values from the entire data set varied over a wide range (−0.5 to about −6 MPa), but the highest (least negative) values exhibited a pattern of decline with increasing VPD that was similar to the previously reported *Prunus* baseline (McCutchan and Shackel, 1992; Figure 1). Overall, SWP values in Spain tended to be closest to the *Prunus* baseline, but some SWP values from other countries were also close to this baseline. The maximum midday air VPD in this data set was about 6.5 kPa, which allowed for a total of 13 groups of 0.5 kPa classes in VPD, having a mode of 2.5 kPa (inset, Figure 1). Because there were relatively few SWP values in each VPD group below 1.5 and above 3.5 kPa (inset, Figure 1), in order to obtain a comparable upper fraction sample from every group, only data from the five central VPD groups (1.5–3.5 kPa) were further analyzed. A regression analysis of average SWP on average VPD for the upper 0.02–0.16 fractions (2–16%) of SWP values exhibited a relatively linear decrease in SWP with increasing VPD regardless of the fraction selected (Figure 2). As expected, selecting greater fractions of the upper SWP values in each VPD group resulted in a progressive decrease in the regression intercept, but no clear trend was apparent in the regression slope (Figure 2). For all fractions from 0.02 to 0.16, the regression *R*^2^ was maximum and the *P*-value minimum for fractions of 0.07 and 0.08, with a clear decline in *R*^2^ and increase in *P*-value as fractions increased above about 0.09 (Figure 3). It should be noted that these *R*^2^ and *P*-values are only used for purposes of comparison, and even though the total number of observations increased with higher fractions, since the regression analysis was performed on the mean (SWP, VPD) values, the number of points (5) for each regression was constant (as shown in Figure 2). Since a decrease in the regression intercept was expected as the fraction of upper SWP values increased, the relation corresponding to an upper fraction of 0.07 was considered the most appropriate estimate for a linear upper limit of olive SWP to air VPD. The slope and intercept for this relation (−0.18 and −0.34, Figure 2), were similar to those reported for *Prunus* (−0.12 and −0.41, respectively, Figure 1).

### Comparison to Model Data

**FIGURE 4:**
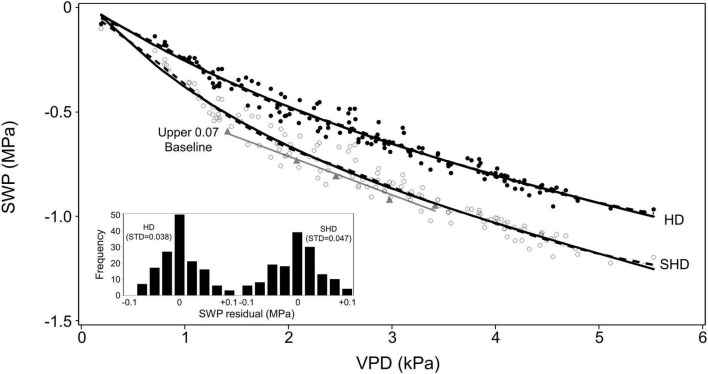
Relation of SWP to midday VPD modeled by García-Tejera et al. (2021) for high density (HD, filled circle) and super-high density (SHD, empty circle) orchard conditions, as well as the same relation for the upper 0.07 fraction of SWP (filled triangles) found in the current study. The dashed lines for HD and SHD models are 50% smoothed spline functions (Proc Transreg SAS 9.4) and the solid lines are a combined linear and exponential function (see Table 2 for parameters) fit to the data. A linear fit (also shown in Figure 2) for the upper 0.07 fraction is shown for reference. Inset shows the distribution and standard deviation (STD) of the residuals from the HD and SHD points to the combined linear/exponential fit. Both distributions were normal.

The soil-plant-atmosphere-continuum (SPAC) model of García-Tejera et al. (2021), which was not based on an explicit link between SWP and VPD, exhibited a clear negative overall relation between SWP and VPD under non-soil-water-limiting conditions, with a similar shape for both HD and SHD orchard conditions (Figure 4). The relation of SWP to VPD was well fit by a smoothed spline function, which involves no *a priori* assumption about the shape of the relation but cannot be easily parameterized, and equally well fit by an exponential decay to a linear dependence of SWP on VPD for both HD and SHD (Figure 4). The residuals to the exponential + linear fit for HD and SHD exhibited a relatively low variation (0.038–0.047 MPa) and a normal distribution (Shapiro–Wilk *P* = 0.37 and 0.74), respectively, and the slope (change in SWP per 1 kPa change in VPD) of the linear component for SHD (−0.13, m in Table 2) was in the same range as that for the strictly linear olive (−0.18, Figure 2) and *Prunus* (−0.12, Figure 1) baselines. One conceptual advantage of the García-Tejera et al. (2021) model over a strictly linear model is that it allows SWP values to approach 0 as VPD’s approach 0, which would be expected for non-soil-water-limited conditions. The relation of SWP to various temperatures and relative humidities for the exponential + linear fit of the García-Tejera et al. (2021) SHD model is presented in Table 3.

**TABLE 2:** Parameters and fit statistics for combined linear + exponential fit shown in Figure 4.

Density	Equation parameters				Fit statistics		
	A	B	C	m	TSS	Model SS	Fit
HD	0.556	2.47	0.0266	−0.096	6.81	6.59	0.97
SHD	0.613	1.37	0.0685	−0.13	9.57	9.25	0.97

$SWP=m*VPD+C-A*\left(1-e^{\left(-\frac{VPD}{B}\right)}\right)$.

**TABLE 3:** Baseline SWP (MPa) for various combinations of air temperature and relative humidity, based on the equation and parameters for SHD density shown in Table 2.

Air temperature (°C)	Air relative humidity (%)					
	10	20	30	40	50	60
5	−0.30	−0.27	−0.23	−0.19	−0.16	−0.11
10	−0.41	−0.37	−0.33	−0.28	−0.23	−0.18
15	−0.54	−0.50	−0.44	−0.39	−0.33	−0.26
20	−0.69	−0.63	−0.57	−0.51	−0.44	−0.36
25	−0.84	−0.78	−0.71	−0.64	−0.56	−0.47
30	−1.00	−0.93	−0.86	−0.78	−0.69	−0.59
35	−1.19	−1.11	−1.02	−0.93	−0.83	−0.72
40	−1.40	−1.30	−1.20	−1.10	−0.98	−0.86

**FIGURE 5:**
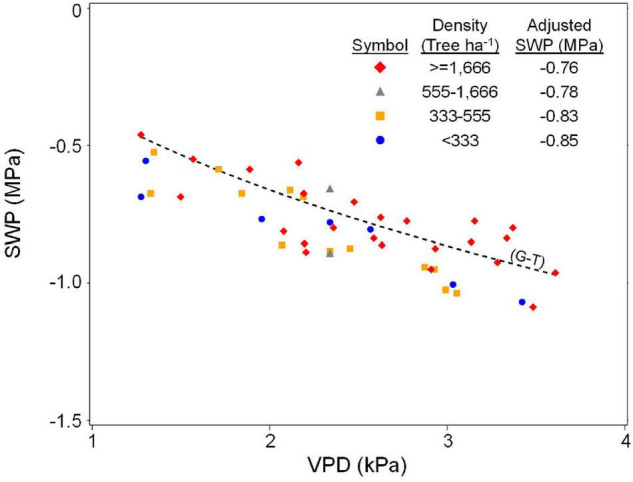
Relation of SWP to VPD for all individual points of the upper 0.07 fraction, classified into groups representing different orchard tree densities, and the adjusted SWP means corresponding to each group. Also shown for reference is the linear/exponential fit for the García-Tejera et al. (2021, GT) SHD model (also shown in Figure 4).

The data used for the olive survey included a wide range of planting densities (Table 1), but the linear estimate for the baseline was much closer to the SHD than to the HD model (Figure 4). All individual survey values that contributed to the upper 0.07 fraction for the linear estimate were categorized based on orchard density, and the least squares mean SWP (i.e., SHD model adjusted mean) for each density was compared (Figure 5). There were no statistically significant differences in the adjusted SWP means from different densities (ANCOVA not shown) but the trend was for an increase in SWP at higher densities (Figure 5), rather than the decrease predicted by the García-Tejera et al. (2021) model (Figure 4).

**FIGURE 6:**
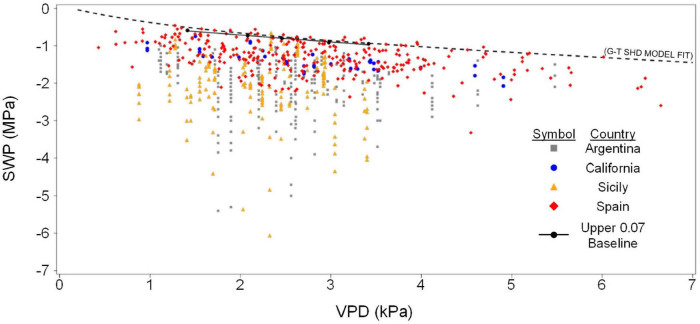
Pooled relation of SWP to midday VPD for all countries, as in Figure 2, showing the combined linear/exponential relation for the SHD data of García-Tejera et al. (2021, dashed line), as well as the points and linear fit for the upper 0.07 fraction found in the current study. Equation for linear fit is SWP = –0.18 × VPD – 0.34.

**FIGURE 7:**
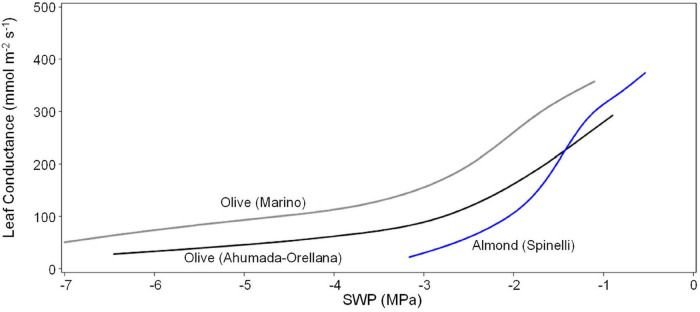
Relation of leaf conductance to SWP for almond reported by Spinelli et al. (2016), for olive reported by Marino et al. (2018), and for olive reported by Ahumada-Orellana et al. (2019). Each line is a 60% smoothed spline function fit to the raw data (Proc Transreg SAS 9.4).

Within the context of the overall range in SWP exhibited by olive under field conditions, the difference between the empirical linear fit and the SHD model fit can be considered relatively minor (Figure 6), with both being surprisingly close to the *Prunus* linear relation (Figure 1). Based on data from the literature, a similar overall relation of Gs to SWP in almond and olive was also found for the upper range of SWP, with close to a linear increase in Gs from about −1.1 to −0.5 MPa in almond and a similar linear increase in Gs from about −1.6 to −0.9 MPa in olive (Figure 7). However, a clear difference between the species was apparent in the lower range of SWP, with almond exhibiting a Gs close to 0 by about −3 MPa, whereas olive maintaining a measurable Gs to about −7 MPa (Figure 7).

## Discussion

As originally proposed (McCutchan and Shackel, 1992), the baseline SWP was intended to serve as a plant-based reference SWP value indicating non-soil-water-limited (“wet soil”) conditions, rather than a plant-based reference value indicating non-physiologically limiting (“non-stressed”) conditions. For instance, a plant under wet soil (baseline) conditions may exhibit the same SWP at high VPD as does a plant under dry soil conditions exhibits at low VPD. In this case, the baseline predicts that irrigation should cause an increase in SWP for the plant at low VPD, but not for the plant at high VPD. However, it does not predict that the increase in SWP at low VPD will have a meaningful impact on plant physiological activity. Thus, as a reference value for irrigation management under field conditions, observed SWP values at or close to the baseline SWP would indicate that soil water was not limiting and hence that no irrigation was needed. Presumably, irrigation under these circumstances may also have an undesirable negative effect on root health. It is also important to consider baseline SWP in order to avoid problems associated with the use of a simple threshold SWP to trigger irrigation, as considered by García-Tejera et al. (2021). Under field conditions, short term (day-to-day) as well as medium term (weather system) patterns in VPD will result in a range of SWP for any given level of soil moisture. Hence, if a desired or “target” SWP has been established for a particular crop and time of year (e.g., prunes: Lampinen et al., 2001; almond: Stewart et al., 2011), then this target must be considered as having a normal range of variation associated with weather (VPD) conditions. Considering the trend of both SWP and baseline SWP over time is required in order to avoid over-reacting to unusually high or low VPD conditions, especially as the target SWP is approached. Observed SWP typically exhibits changes in parallel with baseline SWP over time even at different irrigation levels (e.g., Figure 5 in Shackel, 2011). Hence, the difference between observed SWP and baseline SWP can be used as a more stable plant-based indicator of any trend in the effects of soil water availability. For instance, it may be possible to combine this trend with a forecasted VPD in order to forecast when SWP will reach a given threshold.

Independently of its use as a baseline index of soil water limitations, SWP itself should be a measure of physiological water limitations, although this assumption has not been without controversy (e.g., Sinclair and Ludlow, 1985). SWP should be mechanistically dependent on multiple physical and biological factors (e.g., water transport properties of the soil and plant, as well as stomatal and atmospheric influences on transpiration), and hence it is somewhat surprising that a similar and relatively straightforward dependence of SWP on VPD should be found for both olive (evergreen) and *Prunus* (deciduous). This, as well as the fact that the baseline relation appears to apply across multiple soil types, planting densities, and environmental conditions, may indicate a convergence of plant adaptations/acclimations related to balancing plant water demand to soil water supply, at least for high levels of soil water availability. In both olive and *Prunus*, the range of baseline SWP is relatively small (to −1.2 and −1.0 MPa at a VPD of 5 kPa, respectively, Figures 1, 4) compared to the range of observed SWP. SWP has been reported to range to about −3 MPa in commercial prune (Shackel, 2011) and almond (Shackel et al., 2010) orchards, and to about −4.5 MPa in almond drought studies (Shackel, 2011). SWP of olives in this and other studies show a somewhat wider range (to about −7 MPa; Marino et al., 2018), and for all of these crops the observed range of SWP should be considered as representative of the crops physiological range. In a number of deciduous crops, reductions in SWP over their physiological range have been closely associated with reductions in various measures of physiological activity such as vegetative (cherry) and reproductive (pear and apple) growth (e.g., Shackel et al., 1997; Naor, 2006) and stomatal conductance and/or photosynthesis (e.g., Spinelli et al., 2016). In fact, most of the above studies have found a nearly linear relation between long term average SWP and long-term average or integrative measures of physiological activity such as final tree or fruit size after multiple or single seasons, respectively. Measures of more dynamic (short-term) physiological properties such as Gs have shown a substantial amount of variability in the level of Gs at any particular SWP, with the relation of Gs to SWP in olive described as exponential (Marino et al., 2018) or segmented linear (Ahumada-Orellana et al., 2019). Using an empirical (smoothed spline) approach, we found that a positive linear trend of Gs with SWP occurred at high SWP in both olive and almond (Figure 7). Since this trend occurred within the baseline range, it may indicate that plant water availability can be physiologically limiting, even if soil water availability is not, but further research will be needed to determine whether these limitations are of any practical importance (i.e., limit plant growth or productivity) for irrigation management. For instance, reductions in Gs due to mild water stress may not affect photosynthesis, but could reduce vegetative growth (Bradford and Hsiao, 1982), potentially resulting in an increase in carbohydrate availability for reproductive processes.

In woody perennials, crop yield and quality are the result of growth, developmental, and biochemical processes that occur over relatively long time frames (seasonal or multi-seasonal). Hence, appropriate target or threshold SWP levels for irrigation management in these crops will depend on which processes contribute to yield and quality at which times, as well as the sensitivity of each process to deficit levels of SWP. Table 4 summarizes the range of SWP for regulated deficit irrigation in olive and the observed crop response during different phenological stages. These threshold values may serve as an approximate guide, but it is recognized that the duration of a given water stress is also likely to be important (Girón et al., 2015; Corell et al., 2020).

**TABLE 4:** Guidelines for the use of SWP for deficit irrigation management in olive trees.

Phenological stage	Response	SWP range	References
**PHASE I**			
Vegetative growth	Maximum growth	At or near baseline, (> about −1 MPa)	Pérez-López et al., 2007; Pierantozzi et al., 2020; Hueso et al., 2021
	Significant growth reduction	−1.0 to −1.2 MPa	Moriana et al., 2012; Gómez del Campo, 2013
	Strong growth reduction	−2 Mpa	Moriana et al., 2012; Gómez del Campo, 2013
Flower/inflorescence development	Maximum inflorescence development and flowering	At or near baseline, (> about −1 MPa)	Rapoport et al., 2012; Hueso et al., 2021
	Significant reduction in flowers and inflorescences	−2 MPa	Beyá-Marshall et al., 2018
Fruit set/endocarp growth	Maximum fruit set	At or near baseline, (> about −1 MPa)	Rapoport et al., 2012
	Little or no effect on endocarp growth	−2 MPa	Gómez del Campo, 2013; Gómez del Campo et al., 2014
	Reduction in endocarp growth and fruit size at harvest	−3 MPa	Gómez del Campo, 2013; Gómez del Campo et al., 2014
	Reduction in endocarp growth and fruit size at harvest; possible effect on the flower induction of next season	Lower than −3 MPa; −4 MPa (predawn stem water potential values)	Gucci et al., 2019
**PHASE II**			
Endocarp schlerification (pit hardening)	No significant yield reduction with rehydration next phase. Negligible fruit drop.	−2 to −3 MPa	Moriana et al., 2003, 2012; Fernández et al., 2013; Girón et al., 2015; Ahumada-Orellana et al., 2017
	Significant yield reduction. Fruit drop.	−3 to −4 MPa	Moriana et al., 2003; Gómez del Campo, 2013; Ahumada-Orellana et al., 2017; Corell et al., 2020
	Fruit shrinkage. Permanent injury to table olives.	Below −4 MPa	
**PHASE III**			
Fruit growth due to cell expansion (table and oil olives)	No significant yield reduction. No significantly lower fruit size.	At or near baseline, (> about −1.5 MPa)	Girón et al., 2015; Corell et al., 2020; Martin-Palomo et al., 2020
Oil quantity and quality (oil olives)	No significant effect on oil accumulation	>−2 MPa	Moriana et al., 2003; Hueso et al., 2019
	Increase in phenolic compounds	Linear increase from −2 to −3 MPa	Sánchez-Rodríguez et al., 2019
	Increase in oil extractability	−3 MPa	Fernández et al., 2011; García et al., 2013
	Decrease phenolic compounds	Below −3 MPa	Sánchez-Rodríguez et al., 2019

*Phenological phases are based on Goldhamer (1999) and Fernández et al. (2013).*

Although differences may occur by region, the irrigation season is commonly divided into three phases in mature orchards. Phase I is the most water-sensitive part of the season because shoot growth and flower development occur. For both processes, irrigation scheduling should be performed such that SWP is near the baseline. Even under such conditions, vegetative growth may not be optimal due to the high water stress sensitivity of growth to reductions in SWP (Pérez-López et al., 2007). In young orchards, crown development is very important for reaching maximum yields per hectare, but in mature orchards with super high densities, moderate water stress (−1 to −1.2 MPa SWP; Moriana et al., 2012; Gómez del Campo, 2013) could reduce pruning costs and increase yields by reducing shading (Trentacoste et al., 2019). Maintaining SWP near the baseline would be the best strategy during these phenological stages because fruit and oil yield is strongly affected by early water stress (Rapoport et al., 2012). However, some evidence suggests that only SWP below −2 MPa in the spring will decrease flower number and its quality (Beyá-Marshall et al., 2018). Allowing trees to reach this level of stress before irrigation could provide significant water savings and allow for a greater number of management options at the farm scale under drought conditions.

Endocarp sclerification occurs during Phase II (Goldhamer, 1999; Rapoport et al., 2012). In this period, low SWP values can be tolerated (−2 to −3 MPa) with minor reductions in yield (Goldhamer, 1999). This too could be important when managing drought impacts at a farm scale.

During the last phase, fruit growth occurs principally due to cell expansion and oil accumulation. In this period, different irrigation strategies for table and oil cultivars are necessary, especially if harvesting is done for green table olives. Fruit size is one of the main quality features of table olives and optimum water status is desirable if previous deficit irrigation has been applied (Girón et al., 2015; Corell et al., 2020). SWP might not be the best indicator for detecting a final effect on fruit size during recovery from water stress because no differences in SWP were related to slight differences in fruit size (Girón et al., 2015; Corell et al., 2020). However, if no previous deficit irrigation has been applied, a moderate water stress could be applied with no fruit size reduction (Martin-Palomo et al., 2020). Oil accumulation is more tolerant than fruit growth to water deficit (Gómez del Campo et al., 2014). Furthermore, optimum water status could increase fruit moisture and decrease oil extractability under commercial conditions (Fernández et al., 2011, 2013; García et al., 2013). Evidence suggests that oil accumulation is not affected until SWP is less than −2 MPa (Hueso et al., 2019). The influence of water deficits on oil quality and sensory characteristics is not completely clear yet, but the best quality oil would be well below the SWP baseline (Grattan et al., 2006; Sánchez-Rodríguez et al., 2019). For example, water deficit often increases total phenols, which is an important component of oil quality.

## Conclusion

Across multiple sites and years, an upper limit of olive midday SWP, presumably corresponding to non-limiting soil moisture (i.e., baseline) conditions in the field, was found to have a negative linear relation with midday air VPD for VPD’s above about 1.5 kPa. This relation was very close (within 0.1 MPa) to that of a recently published olive hydraulic model for non-limiting soil moisture and VPD’s above about 2 kPa. This relation was also remarkably similar across the entire range of VPD’s (0 to 6 kPa) to the SWP baseline in *Prunus*. This similarity between *Prunus* and olive, despite many fundamental physiological differences (e.g., *Prunus* being deciduous and olive being evergreen), may indicate a convergence in woody perennial plant adaptations/acclimations that impact the balance between plant water demand on one hand and soil water supply on the other, at least under high levels of soil water availability. The proposed baseline should serve as a reference for olive SWP under non-limiting soil moisture conditions, and it may be important for irrigation management to maintain trees near this reference during stress sensitive periods (e.g., spring). Tentative SWP guidelines for irrigation management during potentially less stress sensitive periods are also presented.

## Data Availability Statement

The raw data supporting the conclusions of this article will be made available by the authors, without undue reservation.

## Author Contributions

KS, AM, GM, MC, and PS substantially contributed to the conception and design of the study. KS wrote the manuscript with assistance from AM, GM, and PS. KS analyzed the full data set from the four countries. DP-L, MM-P, TC, FPM, LMA, LM, RR, and AF were all involved in field measurements, data processing, and supervision of these tasks in the different countries. All authors contributed to the article and approved the submitted version.

## Conflict of Interest

The authors declare that the research was conducted in the absence of any commercial or financial relationships that could be construed as a potential conflict of interest.

## Publisher’s Note

All claims expressed in this article are solely those of the authors and do not necessarily represent those of their affiliated organizations, or those of the publisher, the editors and the reviewers. Any product that may be evaluated in this article, or claim that may be made by its manufacturer, is not guaranteed or endorsed by the publisher.
